# Acetabular deficiency in borderline hip dysplasia is underestimated by lateral center edge angle alone

**DOI:** 10.1007/s00402-022-04652-6

**Published:** 2022-10-22

**Authors:** Daniel Dornacher, Bernd Lutz, Michael Fuchs, Timo Zippelius, Heiko Reichel

**Affiliations:** Department of Orthopedics, University of Ulm, Oberer Eselsberg 45, 89081 Ulm, Germany

**Keywords:** Borderline hip dysplasia, Tripe pelvic osteotomy, Hip joint preservation surgery, Acetabular coverage

## Abstract

**Introduction:**

In hip preservation surgery, the term “borderline hip dysplasia” was used when the lateral center edge angle (LCEA), historically described by Wiberg, measured 18–25°. In recent years, several radiographic parameters have been described to assess the antero posterior coverage of the femoral head, for example, the anterior and posterior wall index (AWI and PWI). This allowed an increasingly comprehensive understanding of acetabular morphology and a questioning of the borderline definition.

**Material and methods:**

A retrospective review of 397 consecutive hips was performed, all treated with triple pelvic osteotomy (TPO) due to symptomatic hip dysplasia. On all preoperative pelvic radiographs with a LCEA of 18–25°, acetabular index (AI), AWI and PWI were measured. With these values, the hips were categorized into laterally, antero-laterally and postero-laterally dysplastic and stratified by gender. Intra- and interobserver correlation of the parameters was analyzed by intraclass correlation coefficient (ICC).

**Results:**

According to LCEA, 192 hips were identified as “borderline dysplastic”. Based on AWI and PWI, the categorization resulted in 116 laterally dysplastic (60.4%), 33 antero-laterally (17.2%) and 43 postero-laterally dysplastic hips (22.4%). Gender stratification revealed that male acetabula seemed to be slightly more postero-laterally deficient than female (mean PWI 0.80 vs 0.89; *p* = 0.017). ICC confirmed highly accurate and reproducible readings of all parameters.

**Conclusion:**

The rather high proportion of symptomatic hips labelled borderline dysplastic suggested, that there might be substantial acetabular deficiency not recognizable by LCEA. Comprehensive deformity analysis using LCEA, AI, AWI and PWI showed, that 40% of these hips were deficient either antero-laterally or postero-laterally. Male acetabula were more deficient postero-laterally than female.

**Supplementary Information:**

The online version contains supplementary material available at 10.1007/s00402-022-04652-6.

## Introduction

Hip dysplasia is well known to be one of the major causes of the development of a premature osteoarthritis. The pathomechanism is thought to be an overloading of the acetabular labrum with subsequent damage, followed by an unphysiological loading of the acetabular rim, leading to cartilage degeneration [1]. Described as early as 1939 by Wiberg, the lateral center edge angle (LCEA) serves as radiographic parameter to assess the acetabular coverage in the diagnosis of hip dysplasia [2]. Historically, according to the original description of Wiberg, hips with an LCEA < 20° were attributed pathological and hips with an LCEA > 25° normal, whereas hips with values in between were labelled uncertain. This confusing uncertainty has led to the terms “mild” or “borderline” hip dysplasia. In recent years, increasing scientific interest has been directed towards this grey area of borderline hip dysplasia. Previous studies have defined the term “borderline hip dysplasia” with an LCEA between 18 and 25° [3–8]. With the aim of joint preservation, corrective pelvic osteotomies became widely accepted as the treatment of choice since they have shown to improve pain and function in patients with true hip dysplasia, associated with an LCEA < 18° [6, 9, 10]. When LCEA measures in the borderline range, orthopedic surgeons performing pelvic osteotomies are more reserved due to the inherent morbidity of these procedures [11, 12]. With advancements in arthroscopic techniques such as labral reattachment and capsular plication, hip arthroscopy migrated towards the treatment of borderline dysplasia, providing significantly less invasivity. The role of isolated arthroscopy for the treatment of dysplasia-associated intraarticular pathologies has been described. Since long-term follow-up still is missing and the examinations report a varying success rate, the importance of arthroscopy as a stand-alone procedure remains unclear [3–7]. In this context, it has to be stressed, that the definition of “borderline dysplasia”, based solely on LCEA, is a major weakness, especially in arthroscopic examinations. With the present knowledge about the complex three-dimensional interaction of the femoral head and the acetabulum and the well-established radiographic parameters for its analysis, it seems greatly simplified to guide joint preservation surgery with LCEA alone [13–15]. The relationship of acetabular version with osteoarthritis of the hip has already been described by Tönnis and Heinecke in 1999 [16]. The impact of anterior acetabular under- and- overcoverage was understood, but objectifiable radiographic assessment tools have not been established back then. In recent years, parameters to quantify the extent of anterior and posterior coverage of the femoral head were introduced, amongst them the anterior and posterior wall index (AWI and PWI) [14]. In combination with the acetabular index (AI) [17], above mentioned parameters contribute to a comprehensive evaluation of the acetabulum. Despite a lateral coverage with an LCEA between 18 and 25°, according to the term “borderline” dysplasia, considerable anterior or posterior undercoverage might become apparent. In other words, the assessment of acetabular coverage only by LCEA could result in overlooking anterior or posterior undercoverage.

Besides their huge benefits in preoperative deformity analysis, the normal values of AWI and PWI provide guidance for the surgeon in the event of an operation [14]. As the term suggests, borderline acetabular dysplasia comes with subtle or more exceptional undercoverage, making correct acetabular orientation delicate. Hartig-Andreasen et al. reported that in mild dysplasia – in their examination equaling an LCEA of 20–25°—only little reorientation is possible before overcorrection might occur [18].

The objective of this examination is to identify the proportion of hips with the definition of borderline dysplasia in our own patient population, to classify these hips into different categories based on radiographic parameters and to investigate gender-specific differences.

## Materials and methods

A retrospective examination of pelvic radiographs was conducted. All radiographs were acquired in the run-up to triple pelvic osteotomy (TPO). The images were used for preoperative deformity analysis and planning of the correction. Between January 2015 and December 2021, 397 TPOs were performed in our orthopedic department on a total of 324 patients (73 patients treated bilaterally, 276 females (85.2%), 48 males (14.8%), mean age 27 years, range 9–48 years, standard deviation (SD) 8.4 years). The patients were referred to our hospital mainly with the diagnosis of symptomatic hip dysplasia. All pelvic radiographs were conducted in the radiological department of our institution, in supine position with a film-focus distance of 1,15 m, the beam centered between the symphysis and a line connecting the anterior superior iliac spines, both legs fully extended and 15° inwardly rotated. The radiographs were archived in the picture archiving and communication system of our institution (PACS, GE Centricity Universal Viewer Version 6.0, General Electric Healthcare, Chalford St Giles, UK). For this specific examination, merely cases with an LCEA between 18 and 25°, measured on the above-mentioned preoperative pelvic radiograph, were included. In particular, when LCEA was measured in the borderline range, all patients received a conservative therapy trial prior to TPO. This comprised physiotherapy, resistance training and/or proprioceptive training over a minimum period of three months. When the non-surgical treatment did not lead to a satisfactory reduction of hip-related pain, TPO was recommended.

**Fig. 1:**
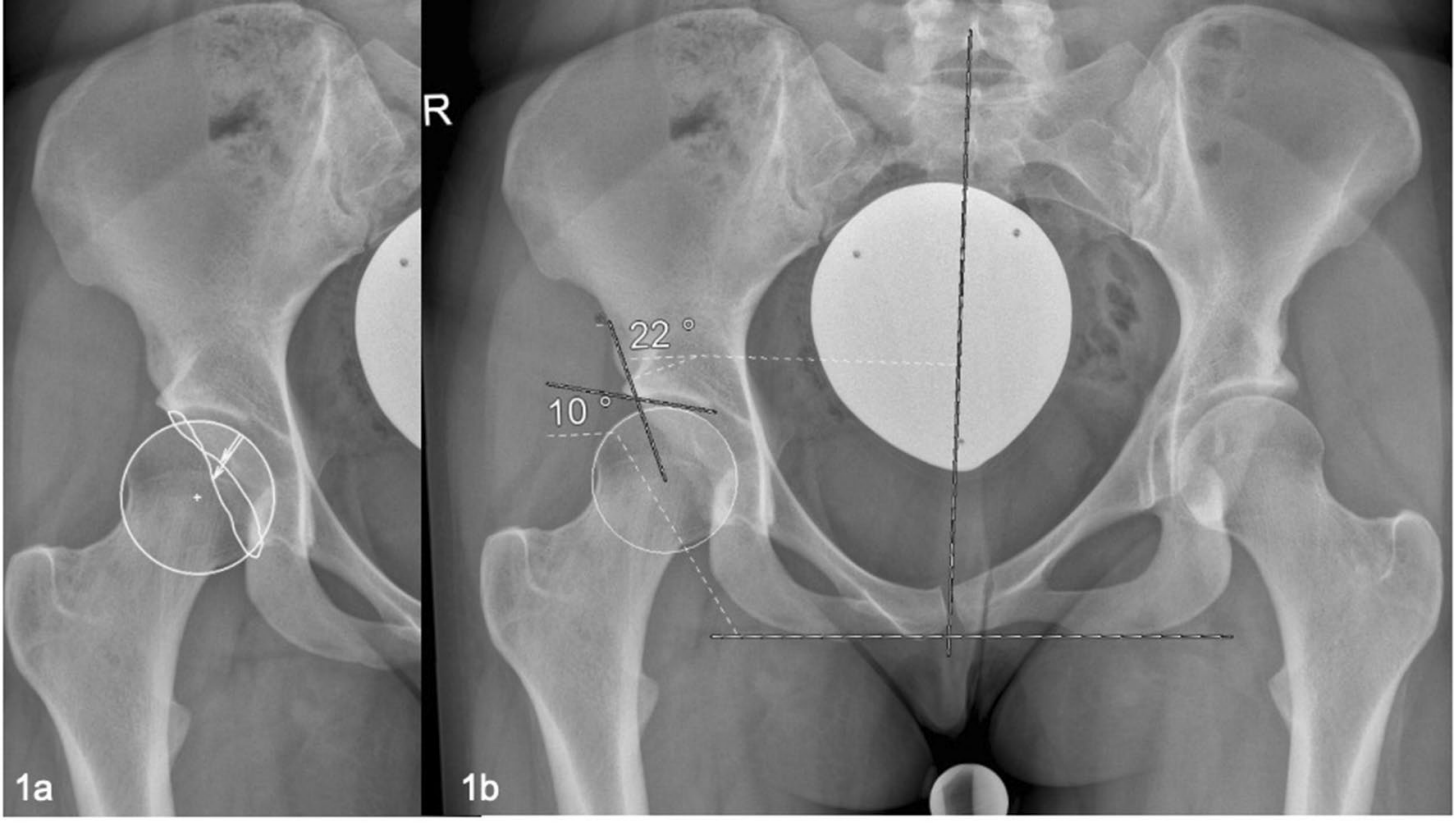
**a** Preoperative pelvic radiograph of a 24 year-old female. The patient complaint about a load-dependent pain radiating towards the groin and the gluteal area. After verification of the usability and the relevant landmarks, first of all, the center of the femoral head was estimated from a circle fit to its contour. For the measurement of the anterior and posterior wall index (AWI, PWI) lines from the medial contour of the circle to its center (white cross, radius = *r*), to the anterior wall (short arrow = *a*) and the posterior wall (long arrow = *p*) were drawn. The distances were measured along the femoral neck axis. AWI and PWI were calculated as *a*/*r* and *p*/*r*. In this example, AWI was calculated 0.41 and PWI 0.70, equivalent with a posteriorly deficient acetabulum. **b** Then, the longitudinal axis of the pelvis was defined by drawing a vertical line from the processus spinosus of L5 through the middle of the symphysis. The LCEA was measured between the line from the center of the femoral head to the lateral aspect of the sourcil, and the longitudinal axis of the pelvis [2, 21]. Acetabular index was measured between a line connecting the inferior ischial tuberosities and a tangent to the most medial and most lateral aspect of the sourcil. In this example, LCEA alone ignored the significant posterior dysplasia

On the preoperative pelvic radiographs LCAE, AI, PWI and AWI were measured by an experienced orthopedic surgeon (observer 1, DD). The measurement routine is displayed in Fig. 1. Power analysis was performed in advance of the assessment of intra- and interobserver correlation (see below). Consequently, the measurements were repeated in 84 randomly selected cases by an experienced orthopedic surgeon (observer 1, DD) and an orthopedic surgeon in training (observer 2, BL).

### Classification into categories

**Fig. 2:**
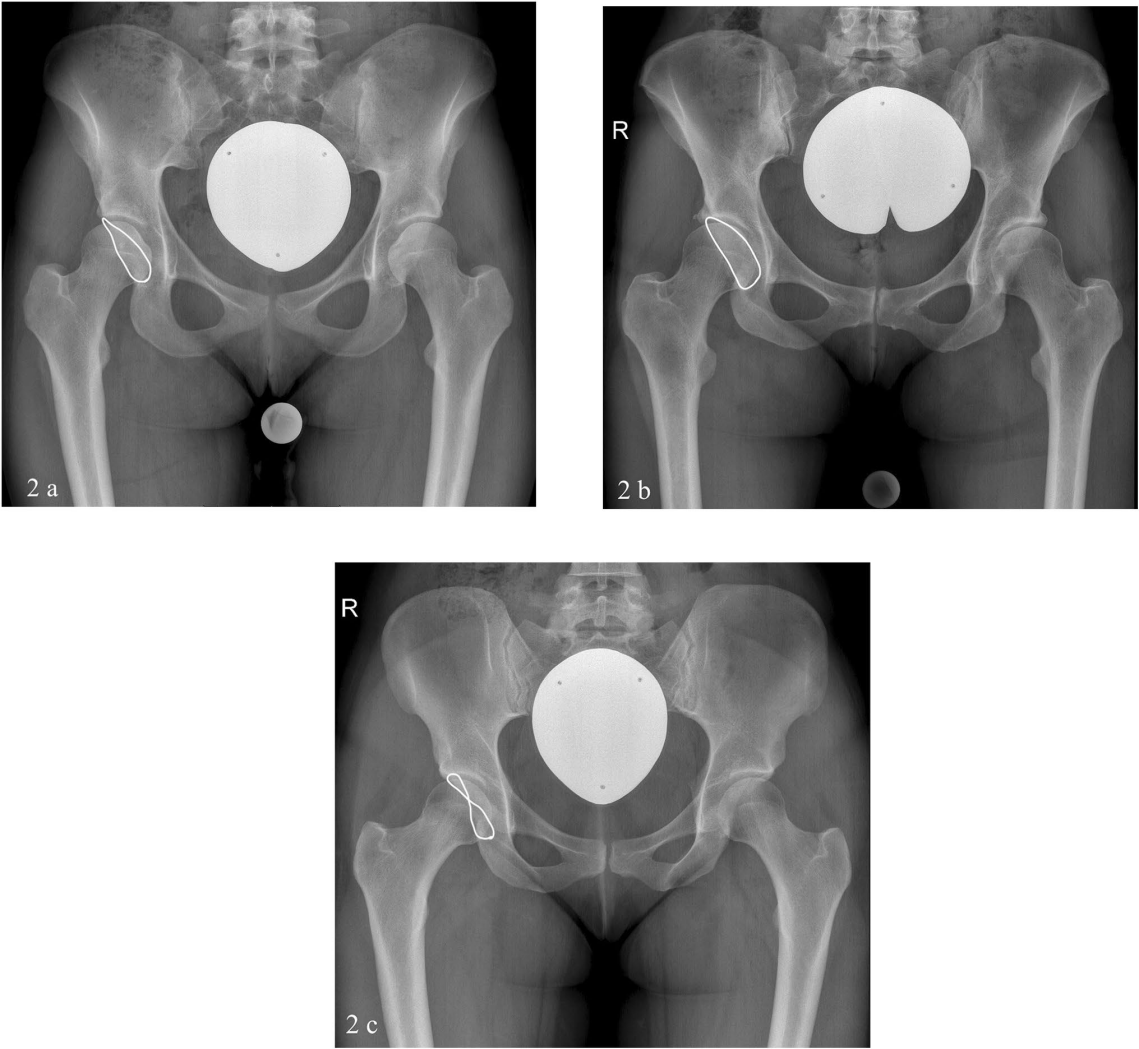
The pelvic radiographs show different types of acetabular orientation in the borderline range. The white line highlights the anterior and posterior acetabular wall. In these examples, acetabular orientation of the right hip was symmetrical to the left one. **a** 28 year-old female, LCEA 20°, AI 10°, AWI 0.32 and PWI 1.00, according to a laterally dysplastic borderline-type. **b** 35 year-old female, LCEA 21°, AI 12°, AWI 0.19 and PWI 0.85, antero-laterally dysplastic type. **c** 26 year-old female, LCEA 19°, AI 13°, AWI 0.51 and PWI 0.51, crossing-sign of anterior and posterior wall, postero-laterally type

According to the values measured in the preoperative radiograph, and taking into account the work of Siebenrock et al. and Bali et al. a classification into three categories was performed [14, 19] (Fig. 2a–c):

Borderline hip dysplasia, laterally deficient (LBHD): LCEA 18–25°, AWI > 0.30, PWI > 0.80; borderline hip dysplasia, antero-laterally deficient (ABHD): LCEA 18–25°, AWI < 0.30, PWI > 0.80; borderline hip dysplasia, postero-laterally deficient (PBHD): LCEA 18–25°, AWI > 0.30, PWI < 0.80.

### Gender stratification

After stratification of the hips into female and male, gender-specific analysis of LCEA, AI, AWI and PWI was performed.

The following exclusion criteria were applied: (1) radiographs with an extraordinarily shallow acetabulum (AWI < 0.30 and PWI < 0.80); (2) radiographs of patients with a severe deformation of the femoral head, e. g. due to Legg-Calve-Perthes disease; (3) radiographs of patients with syndromic diseases.

### Statistical analysis

Intra—and interobserver (observers 1 and 2) correlation of the acetabular parameters measured on the radiographs was assessed using intraclass correlation coefficient (ICC). The 95% confidence interval (95% CI) was calculated. The values of ICC were interpreted according to the scale described by Cicchetti: less than 0.40: poor, between 0.40 and 0.60: fair, between 0.60 and 0.75: good and greater than 0.75: excellent [20].

Gender-specific differences in the values for LCEA, AI, AWI and PWI were analyzed by unpaired *t* test, two-tailed, *α* set to 0.05.

The statistical analysis and presentation were performed using SPSS Statistics, Version 26 (IBM, Armonk, New York, United States of America).

For ICC, a priori power analysis indicated a minimum sample size of 84 cases (power 0.80, *α* set to 0.05, respectively) (G*Power Version 3.1.9.6).

## Results

From the initially 397 hips, 201 (50.6%) met the above-mentioned criteria for borderline hip dysplasia (LCEA 18–25), After application of the exclusion criteria, 192 hips (48,4%) were suitable for further examination: four hips were excluded due to an extraordinarily shallow acetabulum (AWI < 0.30 and PWI < 0.80), three hips were excluded due to a severe deformation after Legg-Calve-Perthes disease and two due to the presence of a syndromic disease.

Finally, the patient population consisted of 169 patients of which 23 were treated bilaterally (156 females (92.3%), 13 males (7.7%), mean age 27 years, range 14–46 years, SD 7.9 years).

**Fig. 3:**
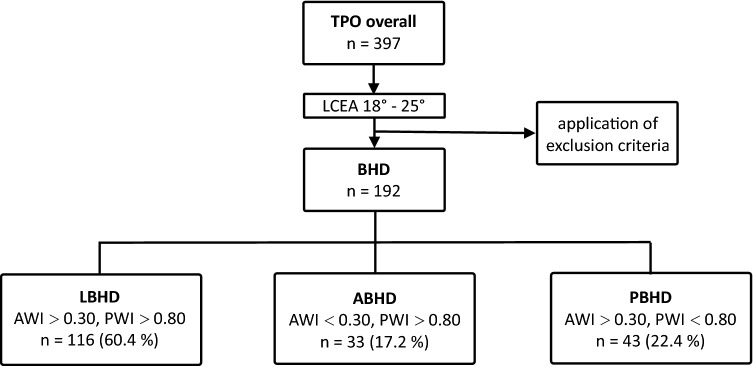
Acquisition of the cases (*TPO* treated by triple pelvic osteotomy) and classification into three categories (*BHD* borderline hip dysplasia; *LBHD* laterally deficient; *ABHD* antero-laterally deficient; *PBHD* postero-laterally deficient)

**Fig. 4:**
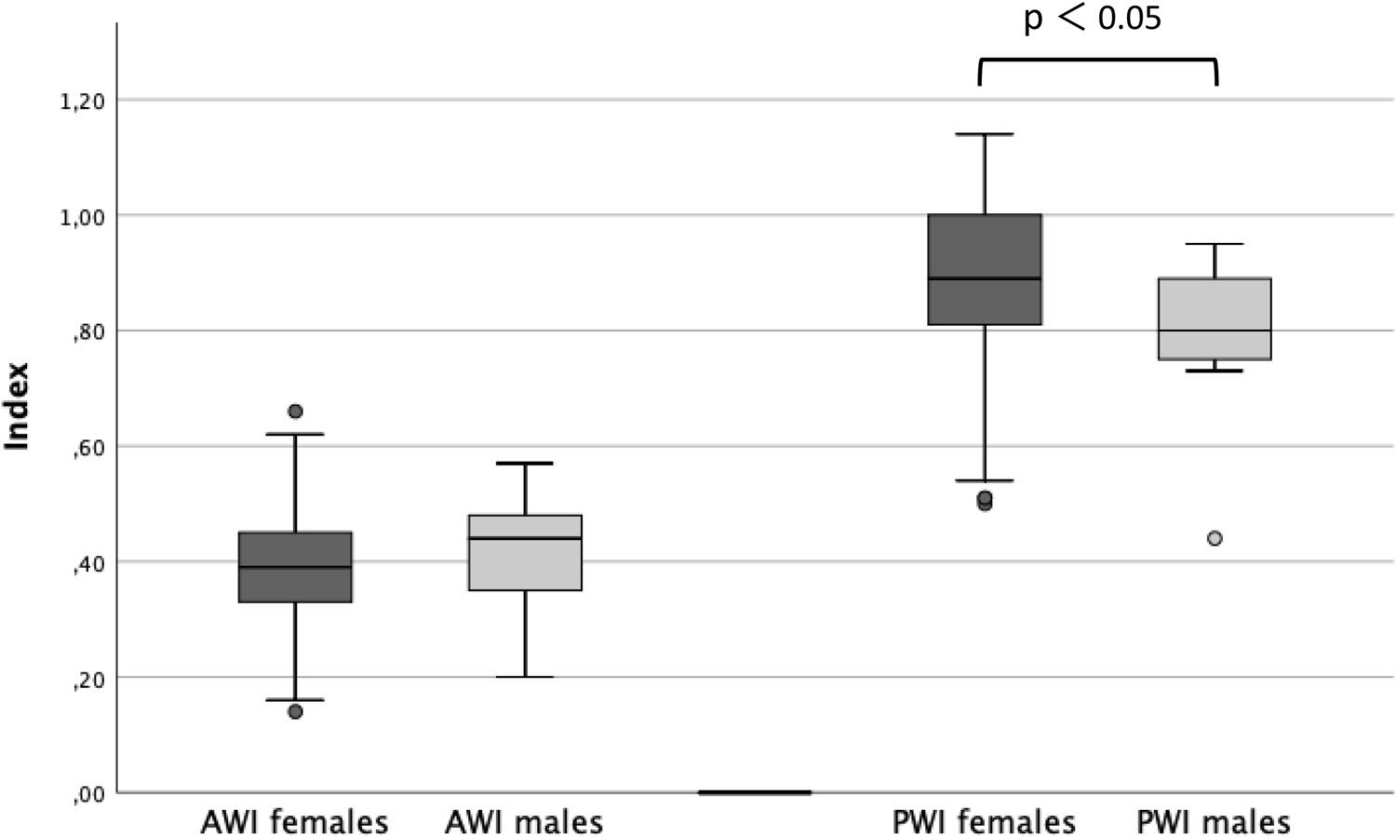
Distribution of the values for AWI and PWI for all hips, stratified into female and male. The boxplots (representing from top to bottom: maximum, 1st quartile, median, 3rd quartile and minimum) show the values for the female (dark grey) and the male hips (light grey). Unpaired *t* test showed a significant difference between the female and the male for the PWI (*p* = 0.017), not for the AWI (*p* = 0.696)

The classification into three different categories (see above), dependent on the preoperative values for AWI and PWI, showed the following distribution: LBHD: 116 hips (60.4%), ABHD: 33 hips (17.2%), PBHD 43 hips (22.4%) (Fig. 3, Table 1). After gender stratification, the following mean values were measured for the females: LCEA: 21° (SD ± 2.2°), AI: 11° (SD ± 4.2°), AWI: 0.39 (SD ± 0.10), PWI: 0.89 (SD 0.13); for the males: LCEA: 20° (SD ± 2.1°), AI 12° (SD ± 3.8°), AWI 0.41 (SD ± 0.12) and PWI 0.80 (SD ± 0.12), respectively. Unpaired *t* test for these parameters showed a significant difference for PWI between females and the males (*p* = 0.017), not for LCAE, AI and AWI (*p* = 0.345–0.696) (Fig. 4), respectively.

**Table 1:** Mean values and ranges of the measured parameters for all cases with borderline hip dysplasia (BHD all), as well as for the laterally (LBHD), antero-laterally (ABHD) and postero-laterally (PBHD) dysplastic hips

	*N* %	LCEA (mean/range)	AI (mean/range)	AWI (mean/range)	PWI (mean/range)
BHD all	192 100%	20.9° (18–25°)	11.2° (0°-22°)	0.40 (0.14–0.69)	0.88 (0.44–0.88)
LBHD	116 60.4%	21.2° (18–25°)	10.8° (0–22°)	0.41 (0.30–0.62)	0.92 (0.66–1.13)
ABHD	33 (17.2%)	20.6° (18–25°)	12.3° (4–21°)	0.25 (0.14–0.29)	0.97 (0.80–1.14)
PBHD	43 (22.4%)	20.5° (18–25°)	11.3° (0–20°)	0.46 (0.32–0.69)	0.70 (0.44–0.79)

Inter and intraobserver correlation analysis resulted in excellent values according to Ciccetti: the lowest values were calculated for the interrater reading of AWI (0.816, 95% CI 0.718–0.879), the highest values for the intraobserver reading of AI (0.988, 95% CI 0.978–0.990) [20].

## Discussion

This study reveals findings with a substantial clinical impact on hip joint preserving surgery: In relation to the overall performed osteotomies, the number of osteotomies performed in hips labelled “borderline dysplastic”, according to LCEA, was considerably high. In this retrospective examination, about 50% of all treated hips were in the borderline range. In 40% of these hips, the radiographic parameters revealed a substantial acetabular deficiency either antero- or postero-laterally.

McClincy et al. conducted a retrospective study of radiographs of their patients which underwent PAO or hip arthroscopy. The respective radiological examinations revealed an LCEA between 18° and 25°. The authors performed a comprehensive analysis to quantify acetabular coverage: LCEA, AI, femoral epiphyseal acetabular roof (FEAR) index, AWI and PWI were measured. Thus, McClincy et al. found a large proportion of dysplastic features among these patients and assumed that an isolated assessment of LCEA was an oversimplistic approach. In conclusion, LCEA was described to be a good marker for lateral coverage of the femoral head, but it failed to encompass other morphologic features of the acetabulum that might contribute to hip instability or FAI, such as focal anterior and posterior undercoverage or unfavorable acetabular version [8].

In our examination, a very similar pattern was recognizable: nearly 40% of the borderline classified hips showed either significant anterior (ABHD, mean AWI 0.25) or posterior acetabular deficiency (PBHD, mean PWI 0.70). In these subgroups, the relevant pathology was not recognizable by LCEA alone.

Bali et al. retrospectively reviewed preoperative pelvic radiographs of their patients who underwent PAO. The authors used LCEA, AI, AWI, PWI, percentage of anterior and posterior coverage as well as the existence of radiographic retroversion signs (ischial spine sign and crossover sign) to classify their hips. This resulted in the “Ottawa classification for symptomatic acetabular dysplasia”, characterizing anterior, posterior and lateral dysplasia. For the latter, the authors detected a significant number without the traditional lateral dysplasia, which could be missed by relying on the LCEA alone. In these hips features of anterior and posterior undercoverage revealed relevant deficiency. The authors went further and stated that the term “borderline dysplasia” was ambiguous and should not be used [19].

In this examination, gender-stratification of the acetabular parameters indicated slightly more postero-lateral deficiency in the male patients (mean PWI 0.80) compared to the female patients (mean PWI 0.89). In presence of an LCEA between 18 and 25°, our results showed no significant difference for the anterior coverage between the genders (mean AWI 0.41 and 0.39, resp).

The work of McClincy et al. demonstrated similar findings: in the borderline range with an LCEA between 18 and 25°, acetabular morphology of female and male patients allowed a distribution in two cluster groups. Therefore, the authors performed separate cluster analyses for each gender. Regarding the acetabular morphology, the females in their study showed rather antero-lateral, the males rather postero-lateral deficiency [8].

Interestingly, in the present study, the proportion of male hips in the borderline subgroup was remarkably smaller (7.7%) than in the entire population (14.8%) including frank hip dysplasia.

This discrepancy might indicate that male patients can better compensate for a borderline dysplastic coverage of the femoral head, whereas female patients more often experience symptomatic micro-instability due to additional ligamentous laxity and less head-centering muscle forces. These “soft factors” were not subject to this examination, but can tip the scale in the decision-making for surgical treatment.

This examination has several limitations: first, this examination focused on acetabular parameters in borderline hip dysplasia. Proximal femoral geometry or femoral torsion was not taken into account. Furthermore, above mentioned “soft factors” in the hip’s anatomy and their contribution to joint stability were not assessed. Second, the vast majority of patients providing the radiographs were female. On 192 patients suitable for this examination, only 13 were male. This has to be put into perspective for the gender-specific statistical analyses. Third, this examination did not consider the clinical outcome of our patients. Although clinical results of patients with borderline hip dysplasia who underwent pelvic osteotomy have been described elsewhere, this will be subject to our future research.

## Conclusion

The high proportion of “borderline” hips in the treatment of symptomatic hip dysplasia suggests that these hips might be mislabeled by the LCEA alone. Comprehensive deformity analysis using LCEA, AI, AWI and PWI showed, that 40% of these hips were substantially deficient either antero-laterally or postero-laterally. There are morphological differences between female and male hips in the borderline range with the male hips being more deficient postero-laterally. In conclusion, it can be stated that LCEA as a single parameter underestimates the true deficiency in borderline hip dysplasia. This has to be taken into account in the deformity analysis and in the decision-making for the treatment.

## Supplementary Information

Below is the link to the electronic supplementary material.

Supplementary file1 (DOCX 29 kb)

## Data Availability

The authors agree to deposit the data that support the findings of this examination. The data have not been uploaded to a public repository yet.
